# Iodine Deficiency Disorder and Knowledge about Benefit and Food Source of Iodine among Adolescent Girls in the North Shewa Zone of Amhara Region

**DOI:** 10.1155/2021/8892180

**Published:** 2021-01-13

**Authors:** Abayneh Birlie Zeru, Mikyas Arega Muluneh, Kassa Ketsela H Giorgis, Mulat Mossie Menalu, Michael Amera Tizazu

**Affiliations:** ^1^Department of Public Health, Institute of Medicine and Health Science, Debre Berhan University, Debre Berhan, Ethiopia; ^2^Department of Midwifery, Institute of Medicine and Health Science, Debre Berhan University, Debre Berhan, Ethiopia; ^3^Department of Nursing, Institute of Medicine and Health Science, Debre Berhan University, Debre Berhan, Ethiopia

## Abstract

**Background:**

As the dietary iodine content depends on the iodine contents of the soil where the crop is harvested, in highland areas where the iodine content of topsoil was washed away due to erosion, iodized salt is the main source of iodine. This study assessed the magnitude of iodine deficiency disorder and knowledge about the benefit and food sources of iodine among adolescent girls in the highland areas of the North Shewa Zone, Amhara Region, central Ethiopia.

**Methods:**

An institutional cross-sectional study was conducted from October 5, 2018, to December 30, 2019. Through a multistage sampling technique, 625 adolescent school girls were selected from 9 schools. A pretested semistructured self-administered questionnaire was used for data collection. Anthropometric measures and thyroid gland physical examinations were performed by trained nurses. The collected data were entered into the computer through Epi Data 3.1 software, and analysis was performed using Anthro plus and SPSS software.

**Results:**

The total goiter rate was 317 (50.7%) with 95%CI of 46.9% to 54.6%. Grade-one and grade-two goiter accounts 226 (36.2%) and 91 (14.6%), respectively. About one-third, 428 (68.5%), had knowledge about locally available iodine source foods and 309 (72.2%) of them mentioned salt as a source of iodine. Over half, 216 (55.1%), of 392 (62.7%) participants who had knowledge about the benefit of iodine knew it prevents goiter. Diet diversity score of <5 food groups [AOR 1.487, 95%CI 1.061–2.083], stunting [AOR 1.876, 95%CI 1.079–3.257], menstruation [AOR 1.615, 95%CI 1.110–2.349], rural residence [AOR 1.412, 95%CI 1.005–1.984], and open salt storage container [AOR 2.001, 95%CI 1.044–3.833] were significantly associated with goiter.

**Conclusions:**

Total goiter rate of adolescent school girls is high in the area. Low diet diversity score, stunting, menstruation, rural residence, and using an open container for salt storage increased the risk of goiter. In addition to universal salt iodization, the emphasis has to be given on proper handling and utilization of iodized salts at the household level to avoid iodine deficiency disorder in adolescent girls.

## 1. Introduction

Adolescence, which is from age 10 to 19 years, is a period of rapid growth of skeletal mass, body size, and body density which highlights the role of nutrients and minerals in the growth process. Due to gender inequality and other physiological needs, adolescent girls are more vulnerable for malnutrition including micronutrient deficiency [1, 2].

Iodine deficiency (ID) is one of those common micronutrient deficiencies during adolescence which results in enlargement of the thyroid gland known as goiter, mental retardation, and physical growth retardation. These clinical and subclinical abnormalities related to ID are termed as iodine deficiency disorder (IDD) [2–6]. But, in adolescent girls, the effect of ID could extend to the fetus and newborns during their motherhood. Mild to moderate ID during pregnancy impairs fetal brain development and reduces child communication skill and, later, school performance and IQ. In addition, it increases the risk of stillbirth, abortions, perinatal deaths, infant mortality, and congenital anomalies [6–8].

To prevent this intergenerational effect of ID, securing adequate iodine intake for adolescent girls before pregnancy is an optimal strategy. Recognizing the importance of preventing IDD, the World Health Organization (WHO) recommended universal salt iodization as the main strategy to achieve the elimination of IDD [9, 10]. Ethiopia started enforcing the comprehensive salt regulation which mandates universal salt iodization in 2012 GC. Since then, iodized salt coverage showed improvement; however, adequately iodized salt coverage is still low [11, 12].

Most IDD-related local studies focused on school-age (6–12 years) children, and those few studies conducted on adolescent girls revealed that goiter is a common public health problem in Ethiopia. The goiter rate among adolescents in Ethiopia varies from place to place ranging from 25.1% to 48.9% [13–15]. Adolescent girls are over two-folds more at risk of goiter than adolescent boys [16]. Grade-one goiter is more prevalent in girls aged 10–14 years, and grade II goiter is more common in 15–19-year-old girls [17].

Because of topsoil erosion where iodine is usually found, there is low dietary iodine content in crops cultivated in Ethiopian highland areas. This means iodized salt is the main dietary source of iodine in those areas [13, 18]. Therefore, it is important to assess the magnitude of IDD and knowledge about the benefits and food sources of iodine among adolescent girls in the highland area of the North Shewa Zone, central Ethiopia.

## 2. Methods and Materials

### 2.1. Study Design and Setting

An institution-based cross-sectional study was conducted from October 5, 2018, to December 30, 2019. Adolescent girls who attend public schools in the North Shewa Zone of Amhara Region, central Ethiopia, were taken as the study population. The North Shewa Zone is one of the eleven zones found in the Amhara regional state. The North Shewa Zone is located at a latitude 9°46'8.4”N and longitude 39°40'4.8”E with an average elevation of 2840 meters above sea level. For administrative purposes, the North Shewa Zone had been divided into 24 districts and 4 town administration units.

### 2.2. Sample Size and Sampling Procedure

The sample size was determined using Epi Info version 7.1.5.0 software by considering 29.3% prevalence of goiter [13], 5% marginal error, 95% confidence level, and a design effect of 2. We got a total sample size of 636.

A multistage sampling technique was employed to get study participants. First, 3 districts (Debre-Sina, Basona werena, and Deneda) from 14 highland districts in the zone were selected using the lottery method. Second, from each selected district, 2 primary schools, rural and urban primary schools, were selected by the lottery method. Since all high schools are located in urban areas, we randomly selected one high school from the selected districts. Three rural primary schools, 3 urban primary schools, and 3 urban high schools, which give a total of 9 schools, were identified for the study. Third, lists of adolescent girls aged from 10–19 years were obtained from each selected school; then, through simple random sampling, the study participants were proportionally recruited from each school for the study.

### 2.3. Data Collection Tools and Procedures

Data were collected by trained female nurses through face-to-face interviews using a semistructured questionnaire and physical examination. The interview was conducted at school during class free time, and it took, on average, 25–30 minutes. The questionnaire was developed by reviewing different kinds of literature and contextually adapted to sociocultural norms and agroclimatic conditions of the study area. The questionnaire was divided into four parts: sociodemographic, dietary practices, anthropometric measurements, and thyroid examination.

Diet diversity assessment: diet diversity was measured by adapting the Minimum Diet Diversity for Women (MDD-W) questionnaire by recalling foods and beverages consumed the previous day and night. The questionnaire was developed by the Food and Nutrition Technical Assistance (FANTA) project to reflect the micronutrient adequacy of women's diets. It has 22 mutually exclusive food groups and categories, of which 14 aggregated to create the MDD-W 10 food group indicator. The ten MDD-W food groups are grains, white roots and tubers, and plantains; pulses (beans, peas, and lentils); nuts and seeds; dairy; meat, poultry, and fish; eggs; dark green leafy vegetables; other vitamin A-rich fruits and vegetables; other vegetables; and other fruits. From those 10 food groups, a minimum of 5 food groups should be consumed in 24 hours to get adequate micronutrients for women. Therefore, a diet diversity score (DDS) of ≥5 was considered adequate [19].

Anthropometric measurement: height was measured to the nearest 0.1 cm with barefoot, and weight was measured to the nearest 0.1 kg with light clothing. Body mass index (BMI) was obtained from WHO Anthro plus software which divides the weight (measured in kg) to the square of the height (measured in meters) of study participants. Similarly, the height-for-age *Z* score (HAZ) and BMI-for-age *Z* score (BAZ) were computed using WHO Anthro plus software. Adolescent girls having an HAZ of <−2 were considered as stunted, and those with <−3 were severely considered stunted. Adolescent girls with a BAZ of <−2 were thin, and those with <−3 were taken as severely thin [20, 21].

Goiter assessment: the enlargement of the thyroid gland related to iodine deficiency was assessed through examination of the thyroid gland. Goiter status was used to assess the IDD of study participants. Those female nurses trained for the data collection also examined study participants for thyroid gland enlargement. Accordingly, the level of goiter was graded as follows:

  Grade 0: if there is no palpable or visible goiter.
  Grade one: if the goiter is palpable but not visible.
  Grade two: if the goiter is visible and palpable under the normal position of the neck according to the WHO recommendation. The total goiter rate of adolescent girls was measured by considering both grade-one and -two goiters. A separate classroom was used for goiter assessment to make girls feel free to show their necks for examination [3, 22].

### 2.4. Data Quality Assurance

The English version of the questionnaire was translated into the local language (Amharic), and the consistency was checked by retranslating back to English and compared with the original version. The Amharic version of the questionnaire was pretested on 25 adolescent school girls to check its understandability by the study participant, response rate to each question, and determine the time required per questionnaire. One-day training on the objective of the study, on data collection tool, and procedure including how to examine the thyroid gland was given for three female data collectors and one supervisor. Daily monitoring and supervision of the data collection and checking for completeness and consistency of collected data were carried out by the supervisor. The principal investigator also examined the collected data for completeness and consistency before data entry.

### 2.5. Data Analysis Procedures

Data were entered into Epi Data 3.1 software and then exported to WHO Anthro plus version 3.1.0 and SPSS version 24 software for analysis. HAZ and BAZ were computed using the WHO Anthro plus software. Descriptive statistics was made to see the sociodemographic characteristics and goiter rate of adolescent school girls. Both bivariable and multivariable binary logistic regression analyses were used to identify factors associated with goiter. Variables with a *p* value of less than 0.25 on the bivariable analysis and nutritionally relevant were considered as candidate variables for further multivariable analysis. Odds ratios (OR) with 95% confidence intervals (95%CI) were computed, and variables with a *p* value <0.05 on multivariable analysis were considered as statistically significant risk factors for goiter.

## 3. Results

### 3.1. Sociodemographic Characteristics

From the total 636 sampled study participants for the study, 625 were involved in the data collection, making a response rate of 98.3%. The mean (±SD) age of study participants was 15.0 (±2.1) years. Two hundred and thirty-three (37.3%) and 156 (25.0%) were in the age categories of 10–14 and 17–19 years, respectively. Over half, 328 (52.5%) and 339 (54.2%), of the participants were primary school attendants and rural residents, respectively. The majority, 224 (37.5%), of mothers of the study participants cannot write and read. The majority of, 363 (59.2%), mothers and, 435 (74.1%), fathers of study participants were housewives and farmers, respectively (Table 1).

### 3.2. Goiter Rate, Nutritional Status, and Dietary Practices

The overall prevalence of goiter was 317 (50.7%) with 95% CI of 46.9% to 54.6%. Of those, 226 (36.2%) were grade-one and 91 (14.6%) grade-two goiter. Seven (1.1%) and 65 (10.4%) participants had severe and moderate stunting, respectively. The overall prevalence of stunting was 72 (11.5%) with 95% CI of 9.1% to 14.2%. From 53 (8.5%) with 95% CI of 6.3% to 10.6% study participants with thinness, 5 (0.8%) had severe thinness and 48 (7.7%) had moderate thinness (Table 2).

Thirty-seven (5.9%) participants had a meal frequency of less than three per day. The mean (±SD) DDS of study participants was 5.01 ± 1.85. Two hundred and eighty-three (45.3%) had consumed less than the minimum required number of food groups (<5 good items) in the last 24 hours (Table 2).

From those 10 food items, grains, 564 (90.2%), were the most frequently consumed food item followed by other vegetables, 508 (81.3%), and pulses, 435 (69.6%). The chi-square test showed that dark green-leafy vegetable consumption showed a significant association with goiter of adolescent school girls (*p* value < 0.001). Grains, white roots and tubers, pulses, other fruits, and other vegetables also were associated with goiter. DDS was significantly associated to goiter (*p* value = 0.005) (Table 3).

### 3.3. Knowledge of the Benefit of Iodine and Iodine Food Sources

Four hundred and twenty-eight (68.5%) study participants believed they knew locally available iodine source foods and the majority, 309 (72.2%), of them mentioned salt as a source of iodine. From 392 (62.7%) participants who knew the benefit of iodine, prevention of goiter, 216 (55.1%), and good health, 178 (45.4%), were more frequently mentioned. From 376 (60.2%) participants who had awareness of salt iodization, 148 (39.4%) mentioned their teachers as a primary source of information. Though most, 568 (90.9%), households of the study participants used closed containers for salt storage, 432 (69.1%) had habits of adding salt at the beginning or middle of the food cooking process (Table 4).

### 3.4. Factors Associated with Goiter

Variables (age, residence, menstrual onset, DDS, type of salt used at the household, and stunting) which had a *p* value <0.25 on bivariable analysis and nutritionally relevant variables such as wasting, meal frequency, and usual salt adding time in the cooking process were included in the multivariable analysis (Table 5).

On the final model, DDS, stunting, menstrual onset, and residence had a statistically significant association with goiter. As compared to those who had ≥5 DDS, the risk of goiter was 1.49 times higher among adolescent school girls who had <5 DDS [AOR; 1.487 and 95%CI; 1.061–2.083]. Stunted adolescent school girls had 87.6% higher odds of having goiter than their counterparts [AOR; 1.876; 95%CI; 1.079–3.257]. The odds of goiter were 61.5% higher among menstruating adolescent school girls compared to their counterparts [AOR; 1.615 and 95%CI; 1.110–2.349]. Rural residents had over 41% higher odds of developing goiter than urban adolescent school girls [AOR; 1.412 and 95%CI; 1.005–1.984]. Using open salt storage containers at household increased the odds of goiter by two-folds [AOR; 2.001 and 95%CI; 1.044–3.833] (Table 5).

## 4. Discussion

In this study, based on the size of thyroid gland enlargement, 36.2% and 14.6% of adolescent school girls had grade-one and grade-two goiter, respectively. The overall goiter rate was 50.7% which reflected that, over half of adolescent school girls were suffering from IDD in the North Shewa Zone highland districts. This is consistent with a 48.9% total goiter rate (36.9% grade I and 11.11.9% grade II) report in Southern Ethiopia [14]. However, it was higher than the report of 39.5% goiter rate among 6–18-year-old girls in Metekel, Northwest Ethiopia [15]. Likewise, it is higher than the 31% total goiter rate among mothers in the highland of the Amhara Region, Ethiopia [13]. This goiter prevalence is much higher than the cutoff point of ≥5% population goiter rate recommendation of the WHO [22, 23] to define IDD as an endemic public health problem. This higher goiter rate in the North Shewa Zone may be related to improper holding and utilization of iodized salt at the household level. Perhaps, it could be due to low dietary iodine intake which is related to the low iodine content of common crops harvested and eaten in the area. The highland areas of the North Shewa Zone are more mountainous and vulnerable for iodine-containing topsoil erosion. As a result, common foods crops harvested and consumed by the community in the area may lack adequate iodine content.

In this study, 68.5% of adolescent girls knew locally available iodine-rich foods which are similar to the 70% report in Northwest Ethiopia [24] and higher than the 61.9% report in Wellega Province of Ethiopia [25]. This study revealed that 62.7% of adolescent girls knew the benefits of iodine and 55.1% of them recognized that it is important to prevent goiter. This finding goes in line with the 2015 national report that two-thirds of women in Ethiopia had awareness about goiter and 48% of them were able to associate goiter with iodine deficiency [26].

MDD-W is one of the best indicators of micronutrient intake adequacy. The current study revealed that the odds of goiter were 1.49 times higher among adolescent school girls who consumed less than 5 food groups than their counterparts, which is in line with the finding of a study in the Dabat district of northwest Ethiopia [27]. This is because when the DDS is high, girls will get adequate nutrients such as iodine for the increased demand during the puberty period. For a population with grains/cereal-based dietary habits, low DDS could lead to iodine deficiency and related disorder of goiter.

Despite the evidence that most packed salts are adequately iodized (≥15 ppm) than unpacked salts and salt iodization is the most effective interventional strategy to prevent IDDs such as goiter [28–30], in this study, the type of salt utilized by the household was not significantly associated with goiter. This could be because of the incorrect timing of salt adding in the food cooking process as over two-thirds, 432 (69.1%), households of adolescent girls in this study usually add salts at the beginning or in the middle of the cooking process. The appropriate time of adding iodized salt to food during cooking is at the end or immediately before the end of cooking to avoid iodine loss through evaporation which reflects the importance of proper handling utilization of iodized salts beside the universal salt iodization to prevent IDD. The level of iodine loss during cooking depends upon the type of cooking procedures and time of addition of salt [31, 32]. Though there is universal salt iodization, because of improper utilization of iodized salts, foods are the main source of iodine in this study area.

This study revealed that postmenarche adolescent girls were 61.5% more at risk of goiter than premenarche girls. According to a study in Switzerland [33] during the mid to late puberty, period girls are more susceptible to goiter even with mild iodine deficiency. This coincidence between goiter and menarche may be related to the sex steroid hormones which promote modulation of the hypothalamic-pituitary-thyroid gland axis. The positive influence of estrogens and minor inhibitory effect of androgens on the circulating thyroid hormone affects thyroid function [34]. This could increase the physiological demand for iodine nutrients during puberty, exacerbate the existing iodine deficiency, and probably lead to the enlargement of thyroid glands in those girls with mild iodine deficiency.

Nutritional status is another risk factor independently associated with goiter. Both goiter and stunting are indicators of long-standing nutritional problems; in this study, stunted adolescent school girls were more likely to have goiter than their counterparts. Likewise, studies in Nigeria showed that stunting is related to goiter [35] and low urine iodine levels [36].

Similar to the finding of a study in the Sawla district of Southern Ethiopia [37], the residence was found to be a risk factor for goiter in the current study. As compared to urban residents, goiter was 41.2% more likely to be observed in rural resident adolescent school girls. The association between goiter and rural residency may be because of a lack of awareness about the cause and preventive methods of goiter which may include improper storage and utilization of iodized salts at the household level.

Despite the large sample size of adolescent girls involved in the study as a strength, this study must have the following limitations: the recent iodine level of study participants was not measured using urinary iodine concentration or iodine content of salt used at the household level; rather, we used total goiter rate which is a less sensitive indicator of IDD. Since the goiter status of study participants was measured through observation and physical examination, there may be nondifferential misclassifications during grading the level of goiter by the data collectors. As a cross-sectional study, the observed association between those identified risk factors and goiter should be interpreted with caution.

## 5. Conclusions

There was a high (50.7%) total goiter rate among adolescent school girls in the North Shewa Zone highland area. Adolescent school girls with low DDS, stunting, menstruation, and rural residency were more likely to have goiter. In addition to universal salt iodization, the emphasis has to be given on proper handling and utilization of iodized salts at the household level to avoid IDD on adolescent girls. The knowledge of adolescent girls about the benefits of iodine-rich foods was low (62.7%), and only half (55.1%) of them had an awareness that iodine-rich foods are important for the prevention of goiter.

## Figures and Tables

**Table 1:** Sociodemographic characteristics of participants in the North Shewa Zone, 2019.

Variables	Responses	Frequency(%)
Age (in years)	10–14	233 (37.3)
	15–16	236 (37.8)
	17–19	156 (25.0)
School	Primary school	328 (52.5)
	High school	297 (47.5)
Residence	Rural	339 (54.2)
	Urban	286 (45.8)
Religion	Christian	603 (96.4)
	Muslim	16 (2.6)
	Others	6 (1.0)
Ethnicity	Amhara	616 (98.6)
	Others	9 (1.4)
Mother educational status	Cannot read and write	224 (37.5)
	Can read and write	219 (36.7)
	Up to grade 8	105 (17.6)
	Grade 9 to 12	24 (4.0)
	College certificate and above	25 (4.2)
Father's educational status	Cannot read and write	85 (14.7)
	Can read and write	300 (51.7)
	Up to grade 8	108 (18.6)
	Grade 9 to 12	48 (8.3)
	College certificate and above	39 (6.7)
Mother's occupation	House wife	363 (59.2)
	Farmer	123 (20.1)
	Merchant	56 (9.1)
	Government employee	31 (5.1)
	Private employee	27 (4.4)
	Others	13 (2.1)
Father's occupation	Farmer	435 (74.1)
	Merchant	49 (8.3)
	Government employee	63 (10.7)
	Private employee	27 (4.6)
	Others	13 (2.2)
Family size	<5	206 (33.0)
	≥5	419 (67.0)

**Table 2:** Prevalence of goiter, malnutrition, and dietary practices of study participants, 2019.

Variables	Responses	Frequency (%)
Goiter	Grade 0	308 (49.3)
	Grade I	226 (36.2)
	Grade II	91 (14.6)
HAZ	Severe stunting (<-3SD)	7 (1.1)
	Mild stunting (<-2SD)	65 (10.4)
	Normal (≥-2SD)	553 (88.5)
BAZ	Severe thinness (<-3SD)	5 (0.8)
	Mild thinness (<-2SD)	48 (7.7)
	Normal (≥-2SD)	572 (91.5)
DDS	<5 food groups	283 (45.3)
	≥5 food groups	342 (54.7)
Meal frequency	≤2 meals per day	37 (5.9)
	3 meals per day	306 (49.0)
	≥4 meal per day	282 (45.1)

Abbreviations: HAZ, height-for-age *Z* score; BAZ, BMI-for-age *Z* score; DDS, diet diversity score.

**Table 3:** Diet diversity and its relationship with goiter among study participants, 2019.

Variables		Responses	Goiter			Chi-square test	
			Yes	No	Total	*χ* ^2^	*p*value
		No. (%)	No. (%)	No. (%)			
Food groups consumed in the last 24 hours	Grains, white roots, and tubers	Yes	275 (86.8)	289 (93.8)	564 (90.2)	8.892	0.003
		No	42 (13.2)	19 (6.2)	61 (9.8)		
	Pulses (beans, peas, and lentils)	Yes	208 (65.6)	227 (73.7)	435 (69.6)	4.828	0.028
		No	109 (34.4)	81 (26.3)	190 (30.4)		
	Nuts and seeds	Yes	176 (55.5)	178 (57.8)	354 (56.6)	0.328	0.567
		No	141 (44.5)	130 (42.2)	271 (43.4)		
	Dairy	Yes	64 (20.2)	68 (22.1)	132 (21.1)	0.335	0.563
		No	253 (79.8)	240 (77.9)	493 (78.9)		
	Meat, poultry, and fish	Yes	58 (18.3)	65 (21.2)	123 (19.7)	0.779	0.377
		No	259 (81.7)	243 (78.9)	502 (80.3)		
	Eggs	Yes	46 (14.5)	50 (16.2)	96 (15.4)	0.357	0.550
		No	271 (85.5)	258 (83.8)	529 (84.6)		
	Dark green-leafy vegetables	Yes	173 (54.6)	211 (68.5)	384 (61.4)	12.799	0.000
		No	144 (45.4)	97 (31.5)	241 (38.6)		
	Other vitamin A-rich fruits and vegetables	Yes	104 (32.8)	112 (36.4)	216 (34.6)	0.874	0.350
		No	213 (67.2)	196 (63.6)	409 (65.4)		
	Other vegetables	Yes	248 (78.2)	260 (84.4)	508 (81.3)	3.924	0.048
		No	69 (21.8)	48 (15.6)	117 (18.7)		
	Other fruits	Yes	59 (18.6)	78 (25.3)	137 (21.9)	4.113	0.043
		No	258 (81.4)	230 (74.7)	488 (78.1)		
	Total DDS out of 10 food groups	<5	161 (50.8)	122 (39.6)	283 (45.3)	7.878	0.005
		≥5	156 (49.2)	186 (60.4)	342 (54.7)		

Abbreviation: DDS, diet diversity score.

**Table 4:** Knowledge about the benefits and food sources of iodine by study participants, 2019.

Variables	Responses	Frequency (%)
Do you know locally available foods used as a source of iodine? (*n* = 625)	Yes	428 (68.5)
	No	197 (31.5)
Iodine source foods (*n* = 428)	Salt	309 (72.2)
	Cereals	81 (18.9)
	Legumes	69 (16.1)
	Fruits	29 (6.)
	Vegetables	57 (13.3)
	Others	15 (3.5)
Do you know the benefit of iodine-rich foods? (*n* = 625)	Yes	392 (62.7)
	No	233 (37.3)
Benefits of iodine (*n* = 392)	Prevents goiter	216 (55.1)
	Good health	178 (45.4)
	Prevent cretinism	84 (21.4)
	Mental development	80 (20.4)
	Physical growth	73 (18.6)
	Prevent abortion or still birth	32 (8.2)
	Others	21 (5.3)
Heard about iodized salt (*n* = 625)	Yes	376 (60.2)
	No	249 (39.8)
Primary source of information (*n* = 376)	Teachers	148 (39.4)
	Mass media	136 (36.2)
	Health workers	106 (28.2)
	Relatives/friends	46 (12.2)
	Others	22 (5.1)
Type of salt used (*n* = 625)	Packed	375 (60.0)
	Unpacked	205 (32.8)
	Both	45 (7.2)
Salt storage container (*n* = 625)	Closed container	568 (90.9)
	Open container	57 (9.1)
Usual time of adding salt during cooking (*n* = 625)	At the beginning of cooking	122 (19.5)
	In the middle of cooking	310 (49.6)
	Immediately before the end of cooking	153 (24.5)
	After cooking	40 (6.4)

**Table 5:** Bivariate and multivariate analysis of factors associated with goiter among adolescent girls, 2019.

Variables	Responses	Goiter		Bivariable analysis		Multivariable analysis	
		Yes	No	COR (95%CI)	*p*value	AOR (95%CI)	*p*value
Age	10–14	109	124	1		1	
	15–16	120	116	1.771 (0.819–1.691)	0.378	0.828 (0.509–1.348)	0.498
	17–19	88	68	1.472 (0.979–2.214)	0.063	0.881 (0.501–1.551)	0.938
Residence	Rural	180	159	1.231 (0.898–1.687)	0.196	1.412 (1.005–1.984)^*∗*^	0.049
	Urban	137	149	1		1	
Menstrual onset	Yes	229	189	1.639 (1.171–2.293)	0.04	1.615 (1.110–2.349)^*∗*^	0.031
	No	88	119	1		1	
DDS	<5 food groups	161	122	1.573 (1.146–2.161)	0.005	1.487 (1.061–2.083)^*∗*^	0.018
	≥5 food groups	156	186	1		1	
Meal frequency	≤twice times day	21	16	1.276 (0.639–2.546)	0.489	1.181 (0.569–2.452)	0.614
	Three times day	153	153	0.972 (0.703–1.343)	0.864	0.809 (0.573–1.142)	0.280
	≥four times day	143	139	1		1	
Stunted	Yes	45	27	1.722 (1.039–2.854)	0.034	1.876 (1.079–3.257)^*∗*^	0.044
	No	272	281	1		1	
Wasted	Yes	28	25	1.097 (0.624–1.927)	0.748	1.359 (0.720–2.563)	0.416
	No	289	283	1		1	
Type of salt used at the household	Packed	179	196	1		1	
	Unpacked	115	90	1.399 (0.994–1.970)	0.054	1.232 (0.831–1.825)	0.081
	Both	23	22	1.145 (0.617–2.125)	0.668	0.844 (0.392–1.814)	0.421
Type of salt storage container	Closed	280	288	1		1	
	Open	37	20	1.903 (1.078–3.359)	0.025	2.001 (1.044–3.833)^*∗*^	0.037
Usual salt adding time	Before cooking starts	60	62	1		1	
	In the middle of cooking	161	149	1.117 (0.734–1.699)	0.606	1.238 (0.766–2.001)	0.678
	Immediately before the end of cooking	77	76	1.047 (0.651–1.685)	0.850	1.383 (0.795–2.405)	0.607
	After cooking	19	21	0.935 (0.457–1.911)	0.573	0.862(0.387–1.911)	0.714

Note: ^*∗*^significantly associated variables at a *p* value <0.05. DDS, diet diversity score; COR, crude odds ratio; AOR, adjusted odds ratio.

## Data Availability

The datasets used and/or analyzed during the current study are available from the corresponding author on reasonable request.

## List of Abbreviations

BAZ: BMI-for-age *Z* score
HAZ: Height-for-age *Z* score
IDD: Iodine deficiency disorder
DDS: Diet diversity score
MDD-W: Minimum siet diversity for women
